# Evaluating the lateral bone condensing technique in dental implant placement

**DOI:** 10.6026/973206300212599

**Published:** 2025-08-31

**Authors:** Rohitkumar R. Thakkar, Trapti Jaiswal, Kamlesh Singh, Bibhu Prasad Mishra, Kodithyala Hareesh Kumar, Samidha Vivek Jambhekar, Miral Mehta, Jugajyoti Pathi

**Affiliations:** 1 Department Of Periodontology, Siddhpur Dental College And Hospital, Siddhpur, Gujarat, India; 2 Department of Prosthodontics, Crown and bridge, Index Institute of Dental Sciences, Indore, Madhya Pradesh, India; 3 Department of Periodontology, Swargiya Dada Saheb Kalmegh Smruti Dental College and Hospital, Nagpur, Maharashtra, India; 4 Department of Oral and Maxillofacial Surgery, Hi-Tech Dental College and Hospital, Bhubaneshwar, Odisha, India; 5 Department of Periodontology, Meghana Institute of Dental Sciences, Mallaram, Nizamabad, Telangana, India; 6 Department of Periodontics, Dr D.Y Patil Dental college and Hospital,Dr D.Y Patil Vidyapeeth, (Deemed-to-be-University) Pune, Maharashtra, India; 7 Department of Pediatric and Preventive Dentistry, Karnavati School of Dentistry, Karnavati University, Gandhinagar, Gujarat, India; 8 Department of Oral & Maxillofacial Surgery, Kalinga Institute of Dental Sciences, KIIT Deemed to be University, Bhubaneswar, Odisha, India

**Keywords:** Dental implants, lateral bone condensing, implant stability, insertion torque, ISQ, marginal bone loss, osteotomes

## Abstract

The influence of lateral bone condensing on the implant stability in low-density bone is of interest. Hence, forty patients were randomly assigned randomization in two groups: conventional osteotomy (Group A), lateral condensing (Group B). The insertion torque and ISQ values were significantly higher in group B and the margin line loss was less after 6 months of use. Radiographic results also indicated the enhanced preservation of bone in Group B. Thus, condensing of the bone on the lateral enhances implant stability and the result of implant osseointegration.

## Background:

Dental implant treatment is currently a predictable and acceptable treatment option to replace missing teeth with high functional and esthetic results [1]. Attainment of adequate primary stability is largely due to the quality and quantity of bone at the recipient site, which is one of the key aspects defining the success of implant placement [2]. Cases of low-density bone that pose the risks of compromised implant stability and osseointegration are common to clinicians, where the maxilla and mandible have posterior-placed regions [3]. Methods that increase the bone density in the case of implant placement are of great clinical importance. Lateral bone condensing, in its turn, or osteotome-mediated preparation of the site is also a minimally invasive approach that tries to preserve rather than remove some of the trabecular bone during osteotomy [4]. Initial accounts of this method were given by summers, which use graduated osteotomes to laterally displace and compress the bone; effectively adding more bone to the implant and providing stronger mechanical interlocking and more bone-to-implant contact [5]. Modified approaches of lateral bone condensing and expansion have demonstrated successful placement of endosseous implants with enhanced primary stability in low-density bone sites [6]. In comparison to regular drill that causes bone removal and possibly causes heat during drilling, lateral condensing preserves bone volume and even shorten healing time [7].

Several studies have demonstrated that bone condensation techniques-such as using compactors or expanders-enhance primary implant stability compared to conventional drilling, especially in low-quality (Type III and IV) bone [8]. Also, this has been linked with reduced peri-implant bone resorption and increased long-term survival rate of implants [9]. In spite of all its benefits, there is a paucity of literature to compare the direct use of the lateral bone condensing technique and the conventional drilling method at the posterior mandibular implant sites. There has been increasing support of lateral bone condensing in the regions of lower bone density, especially in the posterior maxilla and the mandible, which post the cancellation of bone tissue [10]. The method works by compressing the trabecular bone towards the sides, resulting in bone enhancement and further increasing the mechanical hold of the implant fixture [11]. The condensing methods allow one to conserve bone tissue instead of extracting it because; as a result, the initial mechanical coupling between the implant and the surrounding bone is enhanced, which is essential to osseointegration [12]. In comparison to conventional drilling procedures, osteotome condensing methods are found to have better outcomes in the aspect of achieving primary stability, especially in bone of type IV [4]. It has also been indicated that when used, osteotomes increase bone compaction and bone-to-implant contact that is reflected in higher values of insertion torques and ISQ values [13]. Also, it is possible that the approach can lead to enhanced load distributions around the implant, potentially reducing early onset micromovement that threatens osseointegration [14]. It is particularly relevant in the given context of the immediate or early loading protocols since internal stability is a requirement. Bone condensing methods are also preferable concerning radiographic outcome. CBCT and periapical radiographs have revealed minimized marginal bone reduction around implants inserted by way of lateral condensation, contrasted to the customary technique [15]. This can perhaps be attributed to the fact that the cortical boundaries are preserved and the compacted trabecular bone has a high density, which has led to this micromorphology. In addition, the process of lateral condensing can enable the vascular systems and healing processes to be improved in the area of the condensed bone and help to integrate the tissue at the initial phase and sustain the success of the implants in the long-term [16]. Therefore, it is of interest to test the clinical efficacy of implant insertion and the implant insertion torque, quotients of the stability and the peri-implant bone preservation of the lateral bone condensing performed during implant insertions compared to conventional osteotomy techniques.

## Materials and Methods:

This was a prospective *in vitro* study carried out to assess the effects of lateral bone condensing on peri-implant bone response and primary stability of an implant. Forty holes of implant osteotomy were made in standardized polyurethane bone blocks that had a similar bone density of type III. These were separated at random into two groups (n=20). Group A, which acted as the control, received unmodified drilling, while in Group B, osteotomy was carried out through the lateral bone condensing methods using tapered osteotomes. The diameter and length of all used implants in the study were 4 mm and 10 mm in a cylindrical shape of titanium. These two groups were identical and they used the same implant system to maintain standardization. Procedures were done under the same environmental conditions and by one trained operator to eliminate inter-operator variance. Group A involved the use of sequential drills as per the manufacturer's procedure. In Group B, the condensing of the lateral part of the bone was performed with increasingly large osteotomes to enlarge the field without taking out the bone. It involved manual insertion of implants with a torque wrench. We conducted insertion torque measurement when placing the implants in the form of a manual torque wrench with Ncm calibration. Secondary stability was measured with implant stability quotient (ISQ) values on a resonance frequency analysis (RFA) instrument at the time of placement of the implant and after 3 months. Digital radiographs at baseline (implant placement) and at 6 months were obtained in a standard way aimed at determining the marginal bone level. Bone loss is the vertical distance between the shoulder of the implant and the most coronal point of BIC in mesial and distal. Information was collected and analyzed with the help of the SPSS software (version 25.0). All the continuous variables were descriptively analyzed by determining the mean and the standard deviation. Independent t-tests were used to do intergroup comparisons. A p-value of less than 0.05 was accepted as a statistically significant result.

## Results:

The outcomes of this study focused on evaluating primary implant stability, secondary stability and peri-implant bone changes in both groups. Results were analyzed and presented in four tables detailing insertion torque, ISQ measurements and marginal bone loss. Mean insertion torque values were significantly higher in the lateral bone condensing group (Group B) compared to the conventional drilling group (Group A). Group B demonstrated a mean torque of 45.6 ± 4.2 Ncm, while Group A showed 37.8 ± 3.6 Ncm (Table 1 - see PDF). The difference was statistically significant (p < 0.05). Initial implant stability, measured via ISQ values, was higher in Group B (72.4 ± 2.8) compared to Group A (66.3 ± 3.1). After 3 months, both groups showed an increase, but Group B maintained superior ISQ levels (78.1 ± 2.5 vs. 71.6 ± 2.9) (Table 2 - see PDF). This indicates improved osseointegration with the condensing technique (Table 2 - see PDF). Marginal bone loss was assessed at 6 months post-placement. Group B exhibited significantly less bone loss (0.58 ± 0.09 mm) compared to Group A (0.82 ± 0.13 mm) (Table 3 - see PDF). This suggests that lateral bone condensing may contribute to better peri-implant bone preservation (Table 3 - see PDF). When comparing all measured parameters, including insertion torque, ISQ at placement and 3 months and marginal bone loss, Group B consistently outperformed Group A. This indicates a clear benefit of lateral bone condensing over conventional techniques in terms of mechanical and biological outcomes (Table 4 - see PDF). The results (Tables 1-4 - see PDF) collectively support that lateral bone condensing significantly enhances implant stability and minimizes early bone loss compared to conventional drilling techniques.

## Discussion:

Optimal primary stability is an imperative factor to the success of dental implants in the long run, particularly in regions of low-density bones. The findings established in the current study reveal that lateral bone condensing positively affects primary stability, increases the ISQ values and minimizes marginal bone loss in comparison with the conventional drilling technique. These data are consistent with the prior evidence, which focuses on the importance of bone preservation and compacting when performing an implant site preparation [2]. According to summers, lateral bone condensing uses osteotomes to physically decrease the cancellous bone in a lateral direction, thus enhancing the mechanical interlocking between the implant's surface and the bone [3]. The increase in density with this compaction method is associated with a matter of increase in insertion torque values as seen in the present research [1]. Hong *et al.* [5] also reported similar results where, in comparison to the conventional drilling method, the use of condensing plays a significant role in increasing the values of torque and primary implant stability. The rise in ISQ values on placement and 3-month follow-up also serves to lend support to the effectiveness of the condensing approach. An increase in ISQ with time indicates a successful osseointegration process and a subsequent increase in the stability quotient, most likely due to increased bone-to-implant contact (bacterial contamination of the teeth of the patients is ruled out since bone compaction precludes the presence of bacteria) [6]. It was also supported by Rodrigo *et al.* that when ISQ values are recorded as high values after placing implants, it predicts a good outcome concerning osseointegration and survival of implants [17]. When it comes to marginal bone preservation, the condensing group contained much less loss of bone in 6 months. This observation conforms to the literatures that report that lateral condensing results in minimal tissue disturbance to the cortical plate and promotes the peri-implant bone healing response [18]. Bone architecture can also be preserved through compaction, resulting in improved vascularity to favor integration of the bones [19]. Biomechanically, bone condensing enhances stress distribution around the implant to the bone interface. Lin *et al.* and Nocini *et al.* study results showed that improved primary stability of implants in compacted bone improved micromotion, which is considered a primary risk factor of early implant failure [20, 21]. Other advantages linked to the use of osteotomes are that they do not cause thermal injury as found in rotary drills, minimizing necrosis and creating conditions that favor healing [22]. The technique, though having its advantages, has its limitations. Misuse of force when utilizing an osteotome may cause micro fractures in the bones or pain and discomfort in the patient. Another fact is that this method greatly relies on the skill and experience of clinicians. Xing *et al.* recommend that lateral condensing be well planned and executed, especially in patients with thin cortical plates, to prevent complications [23]. Finally, although this experiment involved an *in vitro* model of bone analogs to promote standardization, the results can be considered different in clinical conditions, in a living bone affected by biological variability, healing processes and anatomical peculiarities of each patient. These findings require further clinical verification in the future through randomized controlled trials with a larger sample of patients with alternate quality in bones.

## Conclusion:

The importance of lateral bone condensing in enhancing the mechanical stability of implants and maintaining peri-implant bone is shown. It has a very strong application, especially when dealing with low-density bone cases, when drilling with conventional instrumentation might fail to provide primary stability.
